# Wilson’s disease combined with systemic lupus erythematosus: a case report and literature review

**DOI:** 10.1186/s12883-018-1085-5

**Published:** 2018-06-15

**Authors:** Yun Zhang, Dongmei Wang, Wei Wei, Xuejun Zeng

**Affiliations:** 1 PublicationChinese Academy of Medical Science (CAMS) and Peking Union Medical College (PUMC), Beijing, 100730 China; 2 Department of Neurology, Nanfang Hospital, Southern Medical University, Guangzhou, 510515 China; 3 Department of Internal Medicine, Peking Union Medical College Hospital (PUMCH), Chinese Academy of Medical Science (CAMS) and Peking Union Medical College (PUMC), Beijing, 100730 China

**Keywords:** Wilson’s disease, Systemic lupus erythematosus

## Abstract

**Background:**

Wilson’s disease (WD) is an inherited disorder in which defective biliary excretion of copper leads to its accumulation, particularly in the liver and brain. Systemic lupus erythematosus (SLE) is a multi-system disorder that can manifest in any system. Cases with concomitant WD and SLE, unrelated to treatment with penicillamine, have been rarely reported.

**Case presentation:**

We report a case of a young woman who had typical neuropsychiatric symptoms and laboratory tests results of WD. She also had concomitant massive hematuria and proteinuria, fever, multiple positive autoimmune antibodies, hypocomplementemia, abnormal lumbar puncture findings and evidence of Sjögren syndrome, which are all rare in WD. Hence, we considered the diagnosis of SLE. Tapering of steroid dosage also confirmed the diagnosis.

**Conclusion:**

Wilson’s disease and SLE have varied clinical manifestations. Herein, we reported a rare case in which the two conditions concomitantly existed. In clinical practice, differential diagnosis of the two diseases is necessary for patients with hepatic, neurological, and psychiatric manifestations.

## Background

Wilson’s disease (WD) is an inherited disorder with defective biliary excretion of copper that leads to its accumulation, particularly in the liver and brain. Systemic lupus erythematosus (SLE) is a multi-system disorder that can manifest in any system. We report a case of a young woman who had typical neuropsychiatric symptoms of WD as well as other abnormalities unrelated to WD. The diagnosis of SLE gradually emerged, although cases with concomitant WD and SLE, unrelated to treatment with penicillamine, have been rarely reported.

## Case presentation

An 18-year-old female was admitted to Peking Union Medical College Hospital in November 2016 with the chief complaints of abnormal limb movements and slurred speech for two years, which worsened 20 days ago. Initially, her movements were slower, her hands were clumsy, and she could not speak clearly. In the past 20 days, she gradually developed dysdipsia, unsteady gait, dyskinesia, significantly increased involuntary movements of limbs and fell > 4 times. Cranial magnetic resonance imaging (MRI) showed abnormal signals in bilateral basal ganglia and thalamus. Electroencephalography (EEG) demonstrated diffused 4–6 Hz theta waves. Slit-lamp examination showed Kayser–Fleischer (KF) ring in both eyes. Her serum ceruloplasmin concentration was 0.033 g/L (Normal range: 0.2–0.6). The symptoms progressively worsened, and she had a fever, with temperature between 37.5 and 38.0 °C, without chills, cough or diarrhea. The patient had difficulty in opening her mouth, could only speak one word at a time, and had occasional torsion spasm at the time of admission.

The patient had xerostomia, keratoconjunctivitis sicca, frequent oral ulcers, with no significant weight loss. There was no history of other diseases, but her mother recalled that she talked less, had behavioral changes, abnormal gait, involuntary smile and involuntary movements of all limbs since five years. The parents and older sister did not have similar symptoms. Physical examination revealed that the patient had normal comprehension, with low-grade fever, hepatomegaly, splenomegaly, dystonia, lack of coordination, slight tremor, dysarthria, dysphagia and right side Babinski sign positive.

**Fig. 1:**
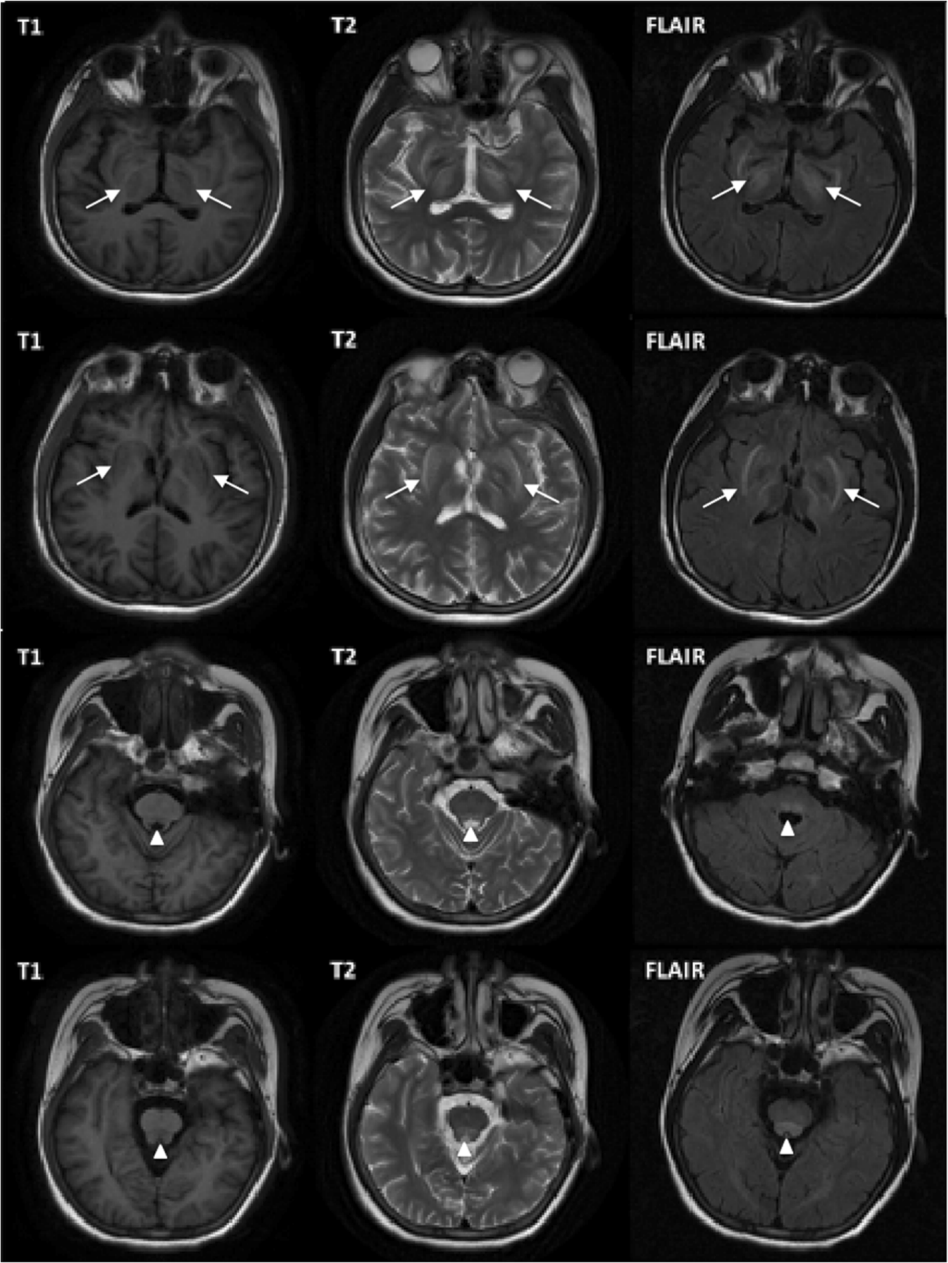
MRI of the patient showed symmetric abnormal signals with low signal in T1-weighted image, and high signals in T2-weighted and FLAIR images of bilateral basal ganglia, thalamus, midbrain, and pons

After admission, routine tests revealed decreased white blood cell count of 2.87*109/L (Normal range: 4–10). Liver function test showed slightly elevated transaminase level and normal bilirubin level. Albumin level was decreased to 32 g/L (Normal range: > 35). Renal parameters were marginally elevated with proteinuria (1.12 g/24 h) and hematuria (++). Lumbar puncture showed elevated intracranial pressure, normal white blood cell count of 2/ul, elevated protein of 0.69 g/L and elevated immunoglobulin G (IgG) of 58.8 mg/L (Normal range: 0–40). Anti-AQP-4 (Anti-aquaporin 4) IgG and myelin basic protein were negative. MRI revealed symmetric abnormal signals with low signal in T1-weighted image, and high signals in T2-weighted and FLAIR images of bilateral basal ganglia thalamus, midbrain, and pons (Fig. 1). Computed tomography (CT) scans revealed diffused lesions in the liver, uneven density, and hepatosplenomegaly. The patient, her parents and her sister underwent genotype test for WD, which showed that the patient had a compound heterozygous mutation, while her family members did not.

Other laboratory findings included elevated ESR, C-reactive protein, IgG, IgM and hypocomplementemia. The titers of antinuclear antibody (ANA) (S1:640), anti-SSA antibody (+++) and anti-rRNP antibody (+++) were remarkably increased, while anticardiolipin antibodies (ACL), Lupus anticoagulants (LA), and anti-β2-glycoprotein-1 (anti-β2GP1) antibodies were all positive. Stomatological and ophthalmological evaluations provided objective evidence of salivary gland (salivary flow rate and parotid sialography) and ocular (Schirmer’s test and ocular dye score) involvement. Salivary gland biopsy showed typical histopathology of Sjögren syndrome. Magnetic resonance angiography (MRA) showed normal arteries, and ultrasound examinations of arteries and veins of bilateral legs, bilateral subclavian, supra-mesenteric, inferior-mesenteric, bilateral renal found no thrombotic evidence.

Based on all the findings, the final diagnosis for this patient was WD, SLE, secondary Sjögren syndrome with anti-phospholipid (aPL) antibodies. Therefore, we started therapy with iv sodium dimercaptopropane sulfonate (DMPS), full dose zinc sulfate for WD, and methylprednisolone (80 mg iv for 7 days, then 40 mg po for 3 weeks), and hydroxychloroquine po for SLE, respectively. Since has three kinds of aPL antibodies were positive, the patient was also treated with anticoagulant therapy (low molecular weight heparin, and then aspirin po). One month later, her neurological symptoms and laboratory tests showed improvement. WBC count, liver function test including transaminase level, bilirubin level and serum albumin level, urine test, ESR, CRP, IgG and complement levels were all normal. Repeat lumbar puncture showed normal parameters. The titer of ANA had declined (S1:160), and aPL (ACL, LA, anti-β2GP1) antibodies were negative. The steroid dosage was gradually tapered and the patient was given oral dimercaptosuccinate (DMSA), zinc sulfate, hydroxychloroquine and aspirin for maintenance.

The patient was followed-up every three months at the outpatient clinic. Six months after discharge, her symptoms recurred, and she developed hyponatremia, hematuria and proteinuria, when the steroid was reduced to 2 mg/day. Hence, methylprednisolone dosage was increased to 16 mg/day, while the other treatment remained unchanged. Symptoms and abnormal laboratory findings were relieved in the next follow-up.

## Discussion and conclusions

**Fig. 2:**
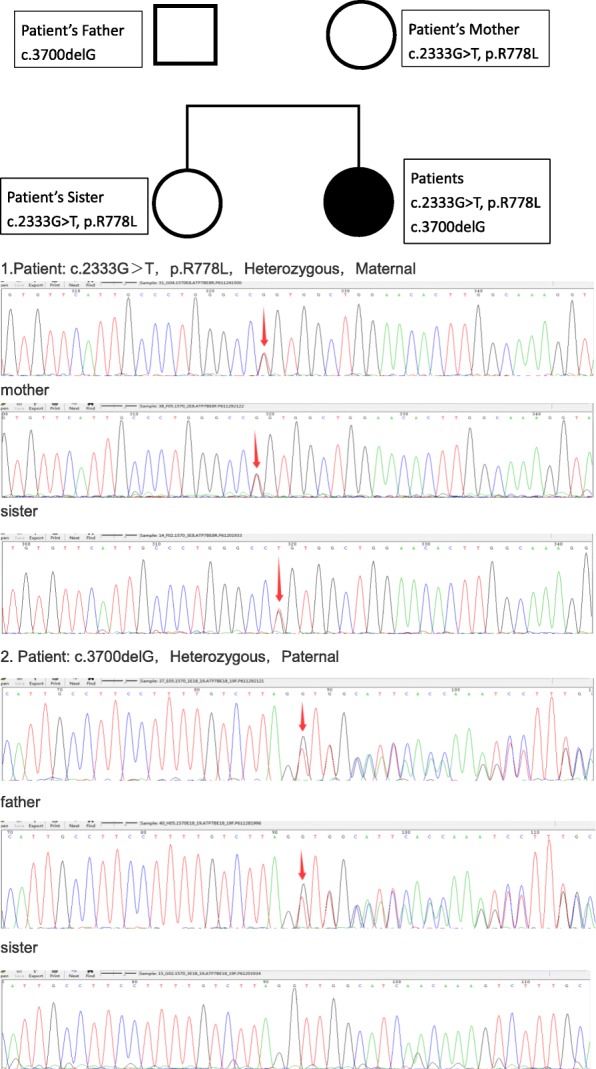
Sequence analysis of ATP7B in the family

Wilson’s disease (WD) is an inherited disorder caused by mutations of the ATP7B gene on chromosome 13, which encodes a copper-transporting P-type ATPase (ATP7B) residing in the trans-Golgi network of hepatocytes. Our patient had a compound heterozygous mutation of ATP7B (Fig. 2), while her parents and sister did not. The clinical features of WD may include hepatic, neurological, and psychiatric manifestations. More than 90% of patients with neurological manifestations have a KF ring [1–3].

Our patient first showed symptoms of abnormal behavior several years before diagnosis. Literature review indicated that psychiatric problems can be the earliest manifestation in adult patients. In addition to the three main movement disorders including dystonia, lack of coordination, and tremor, our patient also presented some common neurological features of WD, such as dysarthria and dysphagia. MRI and CT scans showed typical findings of WD. However, lumbar puncture revealed increased pressure, increased protein level and immunoglobulin level, which were unrelated to WD. Moreover, our patient had massive hematuria and proteinuria, which are rare in WD. Because of fever, multiple autoimmune antibody positive, hypocomplementemia, renal involvement, abnormal lumbar puncture findings and evidence of Sjögren syndrome, we considered the diagnosis of SLE. Tapering of steroid dosage also confirmed the diagnosis.

WD can concurrently occur with autoimmune hepatitis and Coomb’s positive autoimmune hemolytic anemia [4, 5]. SLE has been reported to be associated with WD in patients treated with penicillamine in the form of drug-induced lupus (DILE). DILE has several different features from SLE [6]. But concomitant SLE and WD without penicillamine treatment is rare [7–9]. A possible pathogenetic mechanism of Fas-mediated apoptosis might play a role in autoimmune disease and WD [10]. In such patients, penicillamines should be avoided. According to the guidelines, we used iv DMPS and oral DMSA for this patient, followed by maintainance zinc monotherapy for WD, and 4 mg methylprednisolone and hydroxychloroquine for SLE. The patient has been following-up for 1.5 years, and her liver function and neurological condition continues to be stable [11].

Wilson’s disease and SLE have varied clinical manifestations. Herein, we reported a rare case in which the two conditions concomitantly existed. In clinical practice, differential diagnosis of the two diseases is necessary for patients with hepatic, neurological, and psychiatric manifestations.

## Abbreviations

ACL: Anticardiolipin antibodies
Anti-AQP4: Anti-aquaporin 4
Anti-β2GP1: Anti-β2-glycoprotein-1 antibodies
aPL: Anti-phospholipid
APS: Anti-phospholipid antibody syndrome
CT: Computed tomography
DILE: Drug-induced lupus
DMPS: Sodium dimercaptopropane sulfonate
DMSA: Dimercaptosuccinate
EEG: Electroencephalograph
IgG: Immunoglobulin G
KF ring: Kayser–Fleischer ring
LA: Lupus anticoagulants
MRA: Magnetic resonance angiography
MRI: Magnetic resonance imaging
SLE: Systemic lupus erythematosus
WD: Wilson’s disease
